# Supplementary material for: SERS Signature of SARS-CoV-2 in Saliva and Nasopharyngeal Swabs: Towards Perspective COVID-19 Point-of-Care Diagnostics
Source: Int J Mol Sci. 2023 Jun 3;24(11):9706. doi: 10.3390/ijms24119706 (PMC10253828; doi:10.3390/ijms24119706)
Supplement: Supplementary file 1 [file ijms-24-09706-s001.zip › ijms-2421871-supplementary.pdf]

Supplementary information

SERS Signature of SARS-CoV-2 in Saliva and Nasopharyngeal Swabs: Towards Perspective COVID-19 Point-of-Care Diagnostics

Sylwia M. Berus[a], Ariadna B. Nowicka[a], Julia Wieruszewska[a], Krzysztof Niciński[a], Aneta A. Kowalska[a]*, Tomasz R. Szymborski[a], Izabela Dróżdż[b], Maciej Borowiec[b], Jacek Waluk[a,c], Agnieszka Kamińska[a]

[a]Institute of Physical Chemistry, Polish Academy of Sciences, Kasprzaka 44/52, 01-224 Warsaw, Poland

[b]Department of Clinical Genetics, Medical University of Łódź, Pomorska 251, 92-213 Łódź, Poland

[c]Faculty of Mathematics and Science, Cardinal Stefan Wyszyński University, Dewajtis 5, 01-815 Warsaw, Poland

* Corresponding author: akowalska@ichf.edu.pl

1. Statistical analysis

In the present work, the supervised and classification methods such as PLS-DA, PCA-LDA, SVMC) were performed for establishing classification models for CoV(+) and CoV(-) samples of saliva as well as nasopharyngeal swabs using the commercial Unscrambler® software (CAMO software AS, version 10.3, Oslo, Norway).

Before multivariate analysis, the SERS data were processed using the following steps: (i) smoothing with a Savitzky–Golay filter (Oslo, Norway), (ii) background correction (concave rubber band correction; the number of baseline points was 34 and the number of iterations was 10), and (iii) normalization using OPUS software (Bruker Optic GmbH, 2012 version,

Ettlingen, Germany). The PCA was completed based on the NIPLAS algorithm, validation (random with 20 segments), significance 0.05, and a SERS spectra number of 120.

Number of samples taken for preparation of calibration models and number of external samples subjected to recognition:

- saliva sets: 129 samples for calibration, 20 samples for validation,
- nasopharyngeal swabs: 88 samples for calibration, 16 samples for validation.

Partial Least-Squares Discriminant Analysis (PLS-DA)

PLS-DA is a supervised method that evolved from PLSR algorithm designed to resolve regression problems and additionally aimed at handling classification tasks. Therefore, PLS-DA links procedures such as: (a) reduction of the dimensionality of complex data, and (b) discriminant analysis (samples classification based on the constructed model). To provide pattern recognition information by PLS-DA method, two types of variables must be established. The explanatory variables that stand for the spectral data forming the matrix X and the corresponding response variables of the matrix Y (categorical data - nominal or ordinal that are setting by the user). Initially, such categorical variables (e.g. CoV(+), CoV(-)) are encoded into continuous variables data (e.g. '1' which is CoV(+) and '0' which is CoV(-)). The number of rows of matrix X is the same as for matrix Y and refers to the number of samples. In turn, the number of columns of matrix Y corresponds to the number of established categories. Thus, PLS regression is used to build a model based on matrix X of the predictors and a dummy matrix Y that expresses the class membership, which consequently leads to classification based on the generated prediction values (real-values) [1–5]. The quality of calibration model can be described by figures of merits: R^2_{cal} (R squared in calibration), R^2_{cv} (R squared in cross validation) that pertain to the explained fraction of the original data by the model; RMSEC (root mean squared error in calibration), RMSECV (root mean squared error

in cross validation) that describe the standard deviation between reference and predicted values.

Linear Discriminant analysis (LDA)

LDA is one of the most widely supervised techniques used for classification purposes [6]. This technique aims at finding the features that maximize the between-class variability and minimize the ratio within-class variability so that the separation between classes is the highest. As LDA is suitable to solve task with different complexity, the linear, quadratic and mahalonobis separators can be applied. An important feature of LDA is that it cannot handle high-dimensional, e.g., spectral data. Therefore, other techniques should be used to perform pre-calculations. The most common one is PCA, which reduces the dimensionality of complex data by projecting them into a new coordinate system described by principal components (PCs) and the null space of the within-class data is deleted. Then, the LDA analysis is performed in the simplified subspace thus created [7–9]. Hence, in these studies to perform proper calculations, two-stage PCA-LDA approach was implemented.

Support Vector Machine Classification (SVMC)

SVM is designed for analysis of linear and non-linear data and for regression as well as for classification purposes [10]. The idea of the SVM algorithm is to search for a hyperplane that would effectively separate the analyzed classes by maximizing the margin of separation. The original data are mapped to simpler Kernel space where the supports vectors are found. Support vectors are objects that lie in the borderlines between classes and are designated to describe the separation between them. In turn, margins are determined around the line of separation and ideally margin is free of any object – the separation is called to be “high quality” then. Using the designated margins and support vectors, the SVM establishes the

hyperplane of separation [11,12]. Adjusting the C and gamma parameters is crucial in the stage of determining the optimal hyperplane involving training set samples.

The regularization parameter (C) describes the dependency between the correct classification of training set data and the size of the margin, i.e., high C values indicate a precise classification of as many samples as possible at the expense of a smaller margin; low C values refer to increasing margin, which results in a greater misclassification of trained data.

The Gamma parameter describes the level of influence of individual training set data and affects the shape of hyperplane, i.e., for low gamma values, the samples far away from the separation line have an impact on it while being created; for high values only, samples lying close to the line have impact [13].

Within **the kernel function** four algorithms are available: linear, polynomial, radial basis function, sigmoid. All these parameters as well as kernel function have been established by grid-search.

2. Tables

Table S1. The intensities ratio of a chosen bands between SERS spectra of saliva CoV(+) and CoV(-).

Band (cm^{-1})	Intensity ratio		The differences between intensity ratio
	CoV(+)	CoV(-)	
654/1002	1.3	0.61	0.69
720/1002	4.17	3.3	0.87
1320/1002	1.72	1.32	0.4
1445/1002	2.72	2.25	0.47

Table S2. Statistical parameters of PLS-DA analysis for saliva and nasopharyngeal swabs (NS)

Type of samples	Latent variables	R^2_{cal}	$R(Pearson)_{cal}$	RMSEC	R^2_{CV}	$R(Pearson)_{CV}$	RMSECV	R^2_P	RMSEP	Total variance
Saliva	11	0.70	0.70	0.27	0.69	0.69	0.28	NA	0.54	X 80%, Y 70%
NS	13	0.66	0.66	0.29	0.63	0.63	0.30	0.35	0.40	X=83%, Y=67%

Table S3. The predicted values and standard deviations for saliva samples obtained in PLS-DA analysis. Samples that have been misclassified are marked in grey.

Sample	Predicted value	Standard deviation	Reference
1	0.52	0.26	1
2	1.11	0.22	1
3	0.73	0.16	1
4	0.39	0.27	1
5	0.58	0.19	1
6	0.82	0.29	1
7	0.81	0.64	1
8	0.57	0.18	1
9	0.76	0.24	1
10	0.64	0.15	1
11	0.35	0.34	0

12	0.36	0.36	0
13	0.19	0.33	0
14	1.23	0.30	0
15	0.92	0.41	0
16	0.80	0.22	0
17	0.17	0.45	0
18	0.46	0.33	0
19	0.33	0.54	0
20	0.06	0.42	0

Table S4. Summary of the classification results obtained for PCA-LDA, SVMC for 20 external saliva samples testing on the validation stage. CoV(+) samples are marked in red, CoV(-) samples are marked in green and incorrectly classified samples are bolded.

Samples of saliva	PCA-LDA			SVMC			Actual
	Classification results for single spectra (n_{tot}=15)		Final response	Classification results for single spectra (n_{tot}=15)		Final response	
	Covid (+)	Covid (-)	Classified	Covid (+)	Covid (-)	Classified	
1	13	2	COVID (+)	12	3	COVID (+)	COVID (+)
2	14	1	COVID (+)	15	-	COVID (+)	COVID (+)
3	15	-	COVID (+)	15	-	COVID (+)	COVID (+)
4	15	-	COVID (+)	14	1	COVID (+)	COVID (+)
5	15	-	COVID (+)	15	-	COVID (+)	COVID (+)
6	15	-	COVID (+)	15	-	COVID (+)	COVID (+)
7	15	-	COVID (+)	15	-	COVID (+)	COVID (+)
8	15	-	COVID (+)	15	-	COVID (+)	COVID (+)
9	15	-	COVID (+)	14	1	COVID (+)	COVID (+)
10	15	-	COVID (+)	15	-	COVID (+)	COVID (+)
11	5	10	COVID (-)	**15**	**-**	**COVID (+)**	**COVID (-)**
12	-	15	COVID (-)	**15**	**-**	**COVID (+)**	**COVID (-)**
**13**	**15**	**-**	**COVID (+)**	5	10	COVID (-)	COVID (-)
14	-	15	COVID (-)	-	15	COVID (-)	COVID (-)
**15**	**15**	**-**	**COVID (+)**	-	15	COVID (-)	COVID (-)
**16**	**15**	**-**	**COVID (+)**	5	10	COVID (-)	COVID (-)
**17**	**15**	**-**	**COVID (+)**	-	15	COVID (-)	COVID (-)
18	-	15	COVID (-)	6	9	COVID (-)	COVID (-)
19	1	14	COVID (-)	-	15	COVID (-)	COVID (-)
20	-	15	COVID (-)	-	15	COVID (-)	COVID (-)

Table S5. The intensities ratio of a chosen bands between SERS spectra of nasopharyngeal swabs CoV(+) and CoV(-).

Band (cm^{-1})	Intensity ratio		The differences between intensity ratio
	CoV(+)	CoV(-)	
654/1330	0.84	0.97	0.13
724/1330	2.2	2.1	0.1 (-)
1445/1330	2.2	2.5	0.3

Table S6. The predicted values and standard deviations for nasopharyngeal swabs obtained in PLS-DA analysis. Samples that have been misclassified are marked in grey.

Sample	Predicted value	Standard deviation	Reference
1	0.17	0.34	1
2	0.40	0.31	1
3	0.73	0.28	1
4	0.46	0.30	1
5	0.67	0.19	1
6	1.16	0.17	1
7	0.87	0.42	1
8	0.97	0.37	1
9	0.05	0.36	0
10	0.34	0.30	0
11	0.17	0.61	0
12	0.25	0.57	0
13	0.52	0.23	0
14	0.51	0.22	0
15	0.05	0.42	0
16	0.10	0.35	0

Table S7. Summary of the classification results obtained for PCA-LDA, SVMC for nasopharyngeal swabs. CoV(+) samples are marked in red, CoV(-) samples are marked in green and incorrectly classified samples are bolded.

Samples of nasopharyngeal swabs	PCA-LDA			SVMC			
	Classification results for single spectra (n_{tot}=15)		Final response	Classification results for single spectra (n_{tot}=15)		Final response	Actual
	Covid (+)	Covid (-)	Classified	Covid (+)	Covid (-)	Classified	
1	**2**	**13**	**COVID (-)**	**-**	**15**	**COVID (-)**	COVID (+)
2	**6**	**9**	**COVID (-)**	12	3	COVID (+)	COVID (+)
3	13	2	COVID (+)	15	-	COVID (+)	COVID (+)
4	**6**	**9**	**COVID (-)**	15	-	COVID (+)	COVID (+)
5	15	-	COVID (+)	12	3	COVID (+)	COVID (+)
6	15	-	COVID (+)	15	-	COVID (+)	COVID (+)
7	15	-	COVID (+)	15	-	COVID (+)	COVID (+)
8	15	-	COVID (+)	15	-	COVID (+)	COVID (+)
9	-	15	COVID (-)	-	15	COVID (-)	COVID (-)
10	6	9	COVID (-)	**11**	**4**	**COVID (+)**	COVID (-)
11	-	15	COVID (-)	-	15	COVID (-)	COVID (-)
12	-	15	COVID (-)	-	15	COVID (-)	COVID (-)
13	**15**	**-**	**COVID (+)**	**14**	**1**	**COVID (+)**	COVID (-)
14	**15**	**-**	**COVID (+)**	**15**	**-**	**COVID (+)**	COVID (-)
15	-	15	COVID (-)	-	15	COVID (-)	COVID (-)
16	-	15	COVID (-)	-	15	COVID (-)	COVID (-)

2. Figures

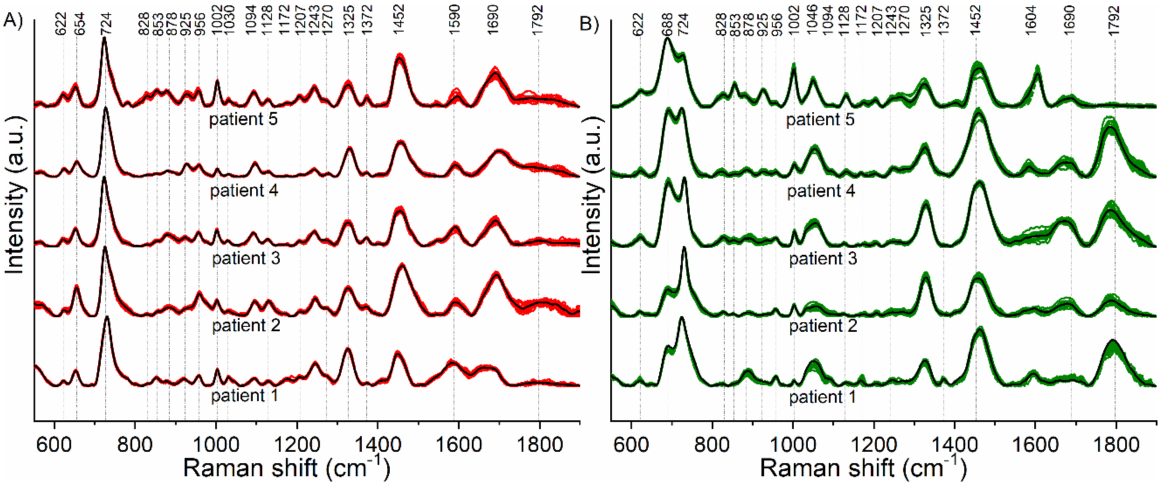

Figure S1. The SERS spectra of saliva recorded for chosen patients infected with COVID-19 (A), a non-infected COVID-19 (B).

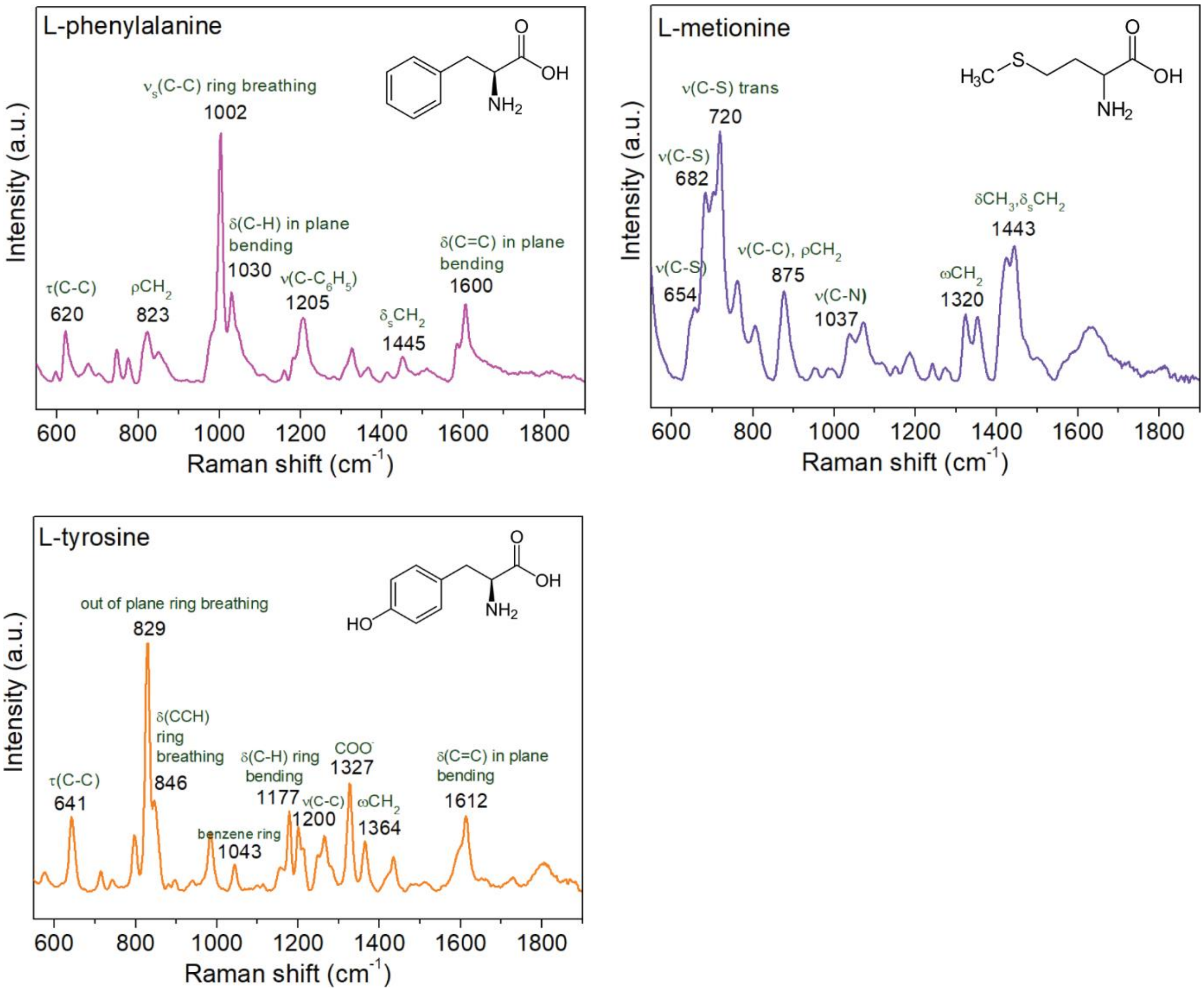

Figure S2. SERS spectra of chosen saline solution amino acids. All the spectra were averaged from 15 single spectra.

Legend: ν, stretching; s, symmetric; as, asymmetric; σ, deformation; ρ, rocking; ω, wagging; τ, twisting/torsion.

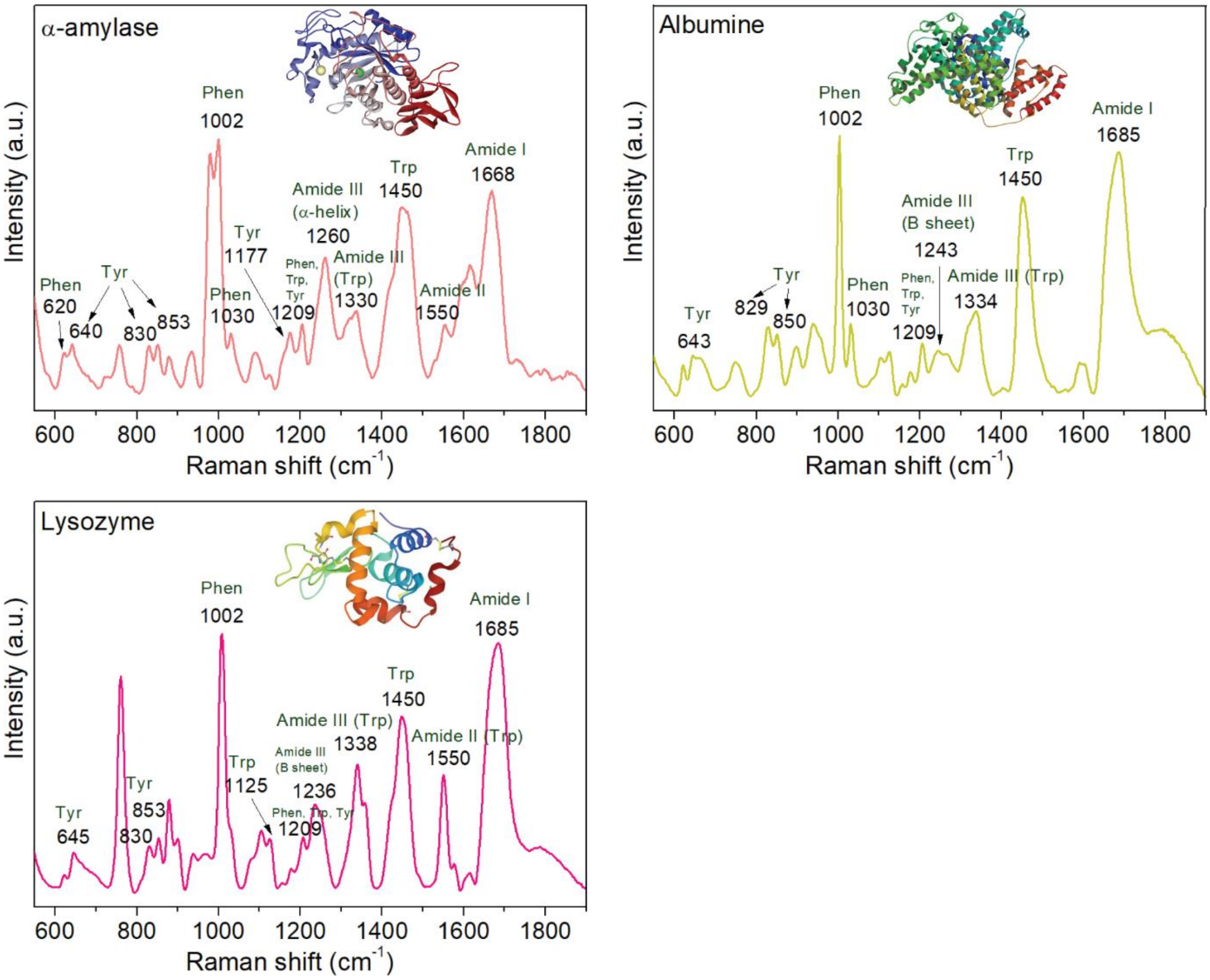

Figure S3. SERS spectra of chosen saline solution peptides. All the spectra were averaged from 15 single spectra.

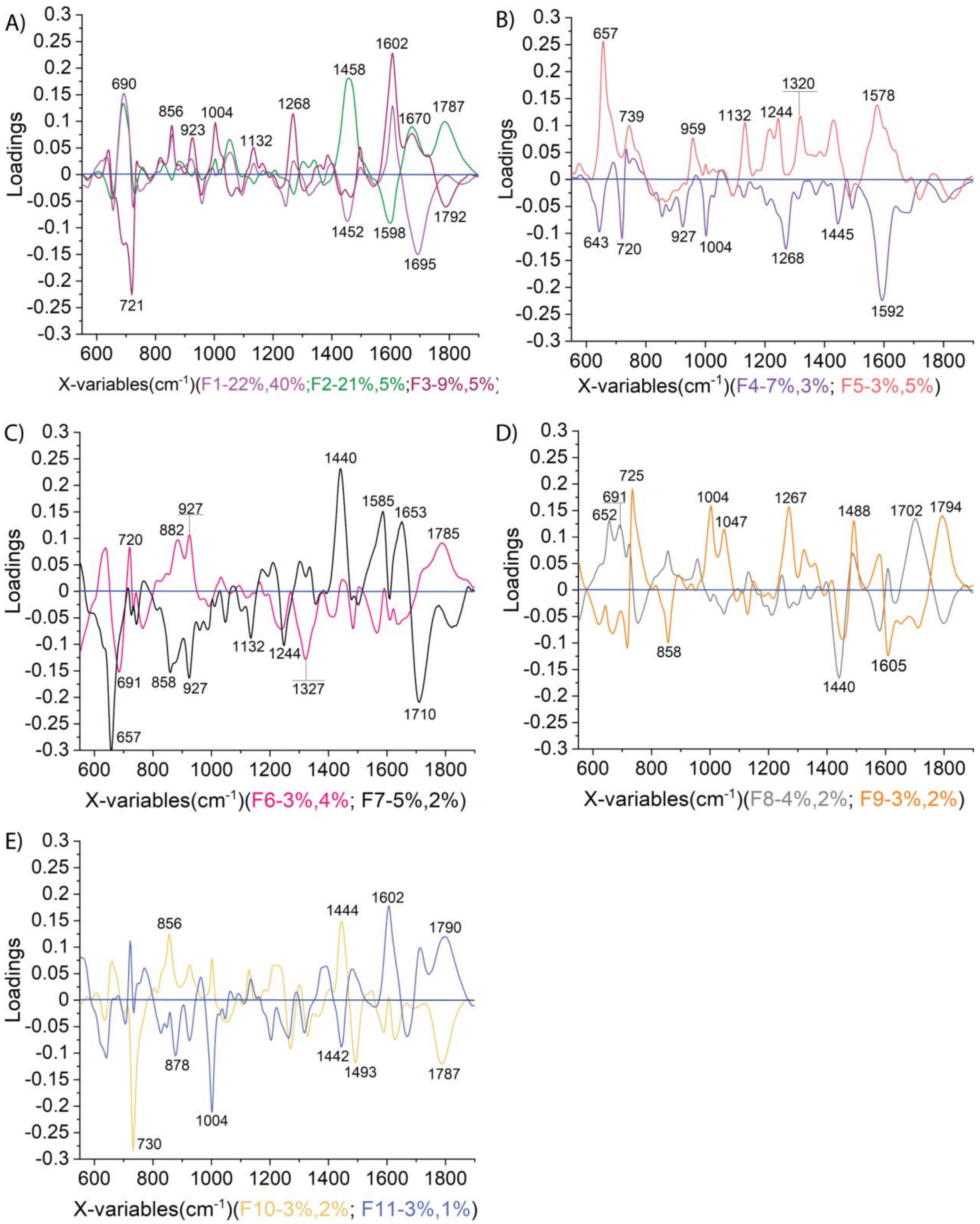

Figure S4a. Loadings plots for the first eleven Factors that are influential according to calculations for saliva samples: **(A)** F1, F2, F3; **(B)** F4, F5; **(C)** F6, F7; **(D)** F8, F9; **(E)** F10, F11. Color coding of the chart corresponds to the labeling of the axis signature (factorial number).

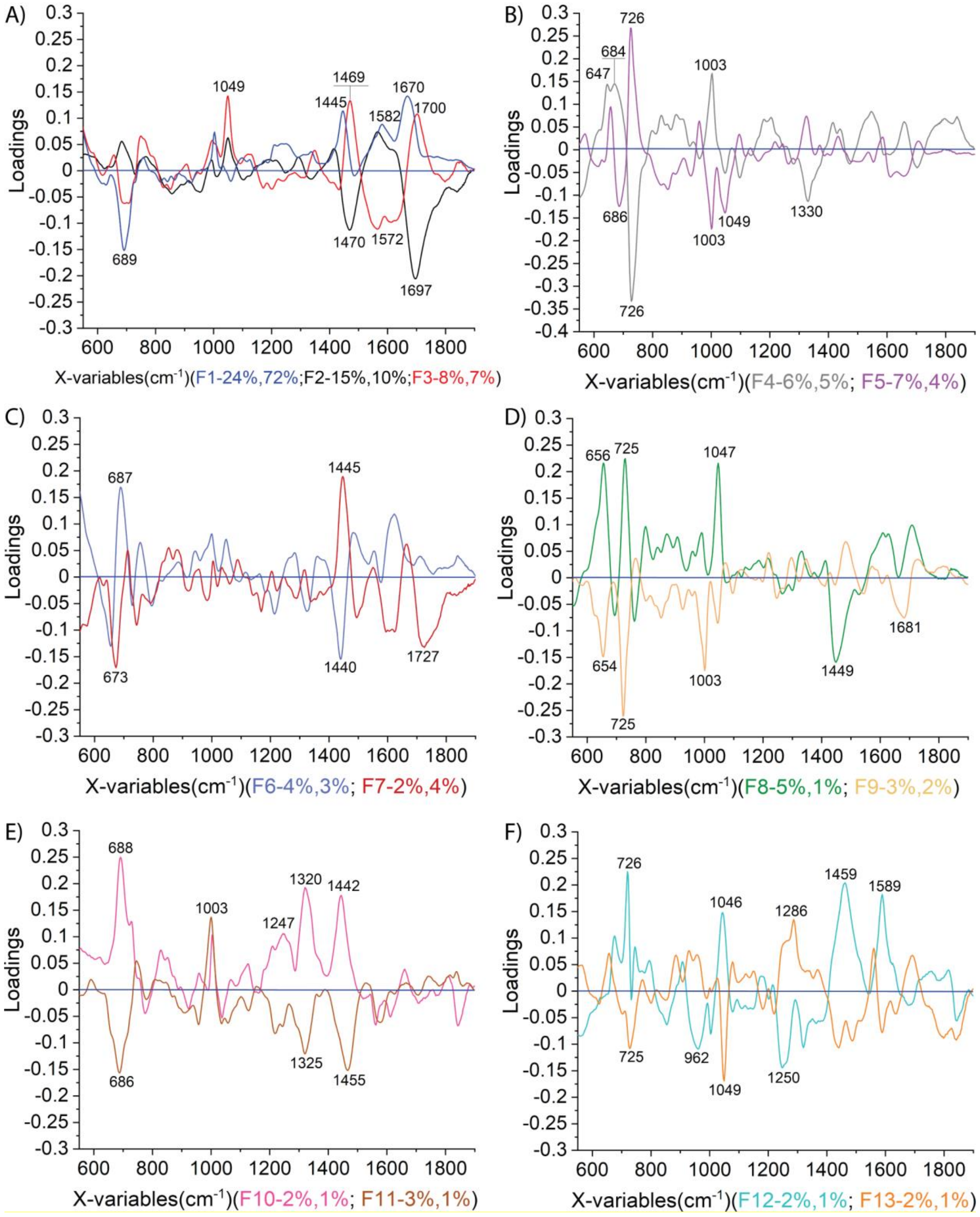

Figure S4b. The loadings plots for the first thirteen Factors that are influential according to calculations for nasopharyngeal swabs: **(A)** F1, F2, F3; **(B)** F4, F5; **(C)** F6, F7; **(D)** F8, F9; **(E)** F10, F11; **(F)** F12, F13. Color coding of the chart corresponds to the labeling of the axis signature (factorial number).

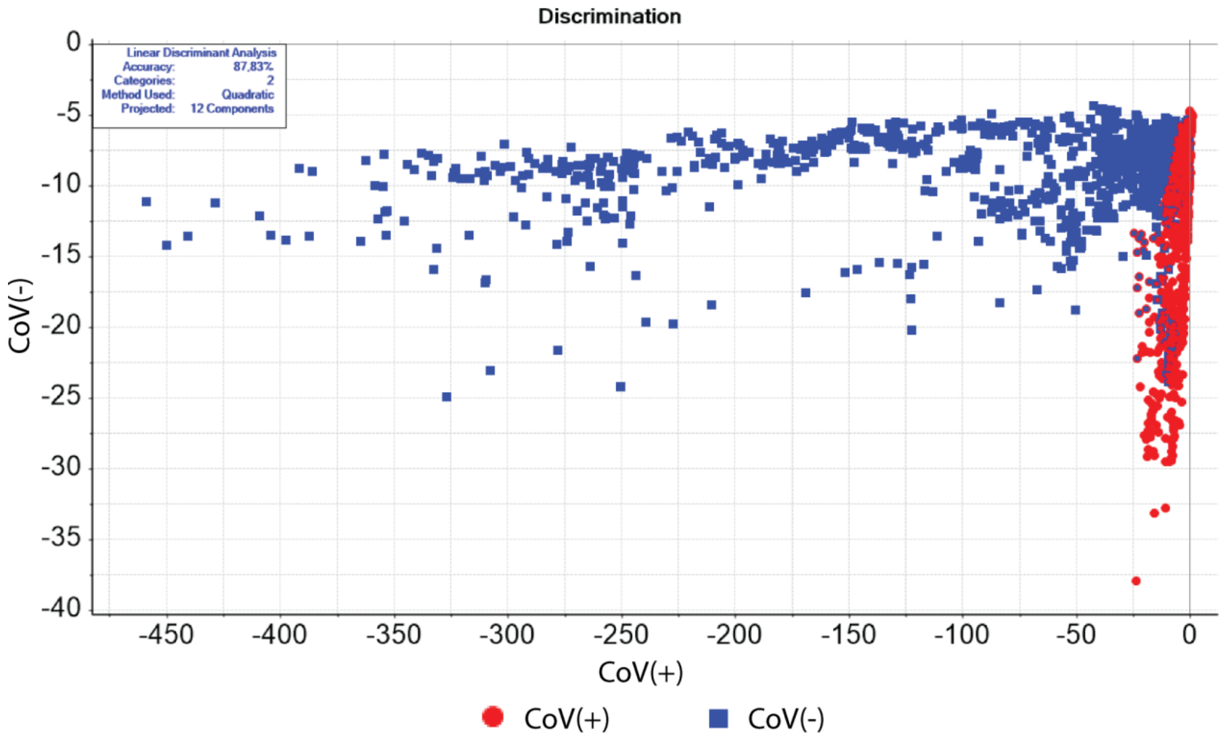

Figure S5a PCA-LDA discrimination plot for the analysis of 77 CoV(-) and 71 CoV(+) saliva samples that creates a calibration model.

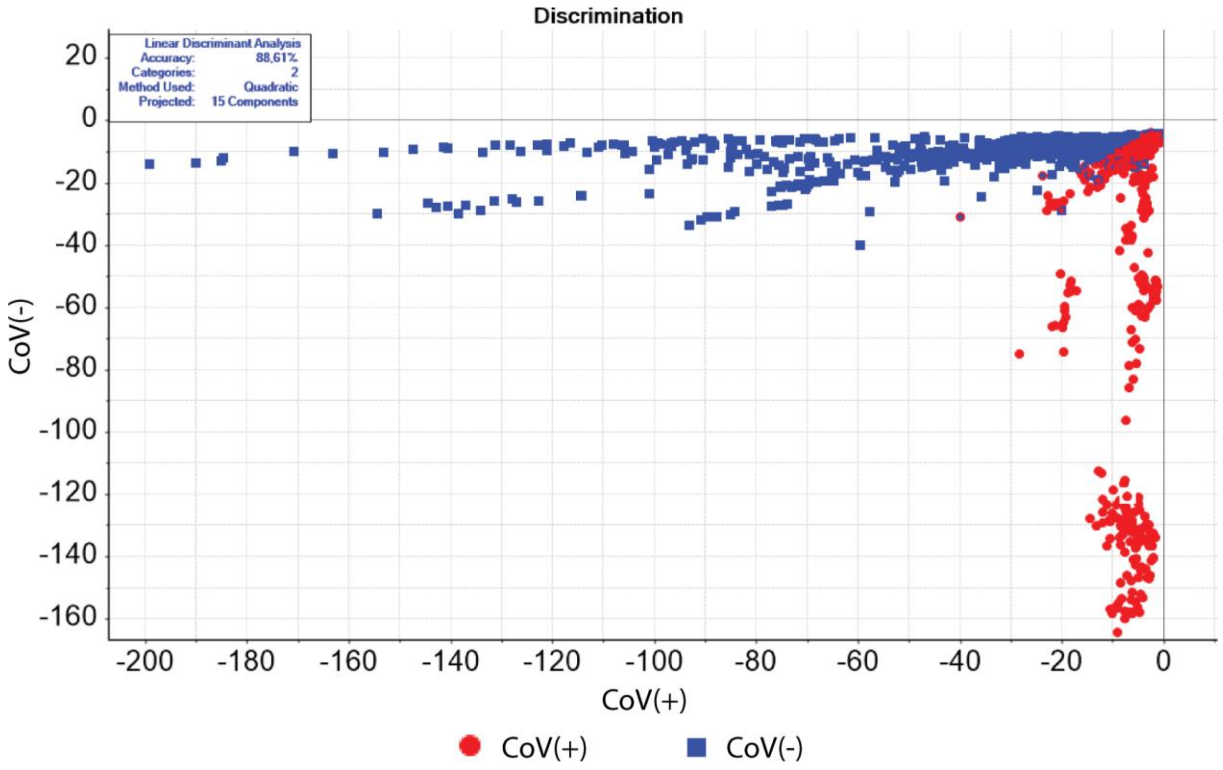

Figure S5b PCA-LDA discrimination plot for the analysis of 53 CoV(-) and 51 CoV(+) nasopharyngeal swabs that creates calibration model.

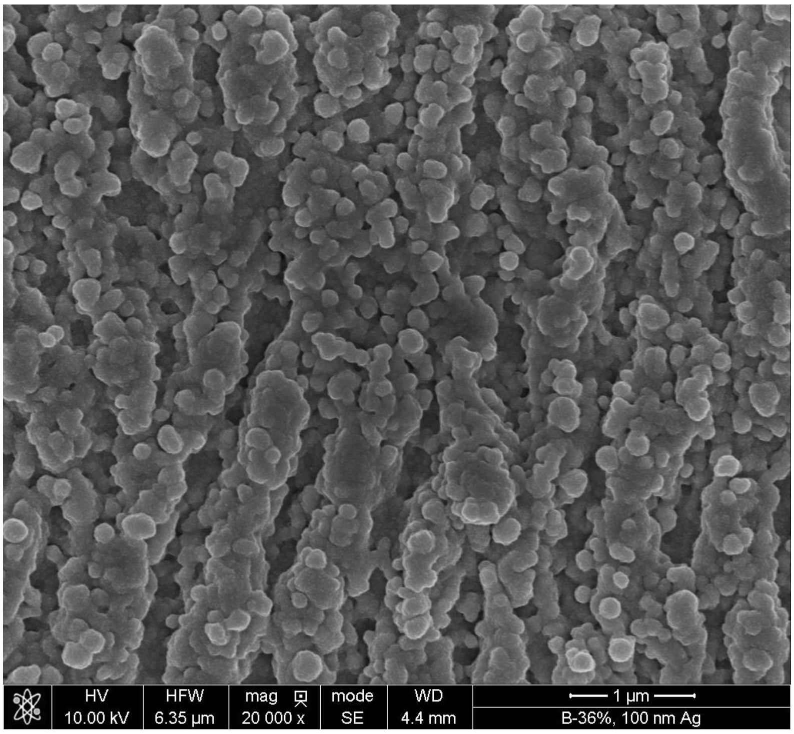

Figure S6. Scanning Electron Microscopy (SEM) image of the surface of the SERS platform. The silicon after laser ablation was covered with 100 nm of silver.

1. Sharma, C.P.; Sharma, S.; Sharma, V.; Singh, R. Rapid and Non-Destructive Identification of Claws Using ATR-FTIR Spectroscopy–A Novel Approach in Wildlife Forensics. *Sci. Justice* **2019**, *59*, 622–629, doi:10.1016/j.scijus.2019.08.002.

2. Bevilacqua, M.; Marini, F. Local Classification: Locally Weighted–Partial Least Squares-Discriminant Analysis (LW–PLS-DA). *Anal. Chim. Acta* **2014**, *838*, 20–30, doi:10.1016/j.aca.2014.05.057.

3. Höskuldsson, A. PLS Regression Methods. *J. Chemom.* **1988**, *2*, 211–228, doi:10.1002/cem.1180020306.

4. Lee, L.C.; Liong, C.Y.; Jemain, A.A. Partial Least Squares-Discriminant Analysis (PLS-DA) for Classification of High-Dimensional (HD) Data: A Review of Contemporary Practice Strategies and Knowledge Gaps. *Analyst* **2018**, *143*, 3526–3539, doi:10.1039/C8AN00599K.

5. Peerbhay, K.Y.; Mutanga, O.; Ismail, R. Commercial Tree Species Discrimination Using Airborne AISA Eagle Hyperspectral Imagery and Partial Least Squares Discriminant Analysis (PLS-DA) in KwaZulu–Natal, South Africa. *ISPRS J. Photogramm. Remote Sens.* **2013**, *79*, 19–28, doi:10.1016/j.isprsjprs.2013.01.013.

6. Suhandy, D.; Suhandy, D.; Yulia, M. Luwak Coffee Classification Using UV-Vis Spectroscopy Data: Comparison of Linear Discriminant Analysis and Support Vector Machine Methods. *Aceh Int. J. Sci. Technol.* **2018**, *7*, 115–121, doi:10.13170/aijst.7.2.8972.

7. Terouzi, W.; Chem, M.J.; Rizki, H.; Kzaiber, F.; Hanine, H.; Nabloussi, A.; Oussama, A. Characterization and Rapid Detection of Adulterations in Sesame Oil Using FT-MIR and PCA-LDA. *Moroccan J. Chem.* **2016**, *4*, 1052–1060, doi:10.48317/IMIST.PRSM/morjchem-v4i4.5167.

8. Yu, H.; Yu, H.; Yang, J. A Direct LDA Algorithm for High-Dimensional Data - with Application to Face Recognition. *J. Pattern Recognitio Soc.* **2001**, *34*, 2067–2070.

9. Lu, J.; Plataniotis, K.N.; Venetsanopoulos, A.N. Face Recognition Using LDA-Based Algorithms. *IEEE Trans. Neural Networks* **2003**, *14*, 195–200, doi:10.1109/TNN.2002.806647.

10. Bordoloi, D.J.; Tiwari, R. Optimum Multi-Fault Classification of Gears with Integration of Evolutionary and SVM Algorithms. *Mech. Mach. Theory* **2014**, *73*, 49–60, doi:10.1016/j.mechmachtheory.2013.10.006.

11. Mohan, V. Liver Disease Prediction Using SVM and Naïve Bayes Algorithms Privacy Preserving Data Mining View Project. *Int. J. Sci. Eng. Technol. Res.* **2015**, *4*, 816–820.

12. Yao, Y.; Liu, Y.; Yu, Y.; Xu, H.; Lv, W.; Li, Z.; Chen, X. K-SVM: An Effective SVM Algorithm Based on K-Means Clustering. *J. Comput.* **2013**, *8*, 2632–2639, doi:10.4304/jcp.8.10.2632-2639.

13. Battineni, G.; Chintalapudi, N.; Amenta, F. Machine Learning in Medicine: Performance Calculation of Dementia Prediction by Support Vector Machines (SVM). *Informatics Med. Unlocked* **2019**, *16*, 100200, doi:10.1016/J.IMU.2019.100200.
